# Cholelithiasis

**Published:** 1904-04

**Authors:** 


### CHOLELITHIASIS.

J. M. G. Carter, in the *American Therapist* for December, 1903, believes that the prophylactic treatment is very important in catarrhal inflammatory affections of the gall-bladder, in fact, that is where the physician's work is most valuable. Some of the cases seem to yield to medical treatment, but perhaps the majority will entirely recover only after operation. It is not always possible for the physician to tell just how his treatment produces a cure. Believing that indigestion, or at least impaired or retarded digestion, has something to do with many of these cases, he directs his treatment towards correcting such trouble, and often prescribes pepsin and aromatic sulphuric acid combined with some laxative. One case recovered after a several months' course of blue-mass once or twice a week. It is well in many cases to combine some vegetable laxative with the mercury. The mercury once a week, and phosphate of soda three to six times a week, do well in other cases. He has used in this class of cases, for several years with beneficial results, a combination of blue-mass, podophyllin, aloin, belladonna and ipecac. Some cases do better on calomel or blue-mass once a week, and small doses of epsom salts three to six times a week. Many cases recover by using two or three ounces of salad or olive oil three to six times a week. In cases in which mercury can not be borne in any form, and epsom salts is objectionable, phosphate of soda and olive oil (taken alternately, each three times a week) may be given. A reduction of meat and fats in the diet is usually beneficial. Especial attention should be given to prevent any degree of constipation. Exercise, moderate diet, avoidance of constipation, and regular habits of life are essential.

